# On an effective and efficient method for exploiting the wisdom of the inner crowd

**DOI:** 10.1038/s41598-023-30599-8

**Published:** 2023-03-03

**Authors:** Itsuki Fujisaki, Kunhao Yang, Kazuhiro Ueda

**Affiliations:** 1 Graduate School of Arts and Sciences, The University of Tokyo, Tokyo, Japan; 2 Faculty of Law, Chuo Gakuin University, Chiba, Japan

**Keywords:** Psychology, Human behaviour

## Abstract

Researchers have shown that even an individual can produce the wisdom of the crowds, called “the wisdom of the inner crowd.” However, the previous methods leave room for improvements in terms of efficacy and response time. This paper proposes a more efficient method, which required a short time, based on findings from cognitive and social psychology. The procedure is to ask participants to give two answers to the same question: first, their own estimate and, second, their estimate of public opinion. Experiments using this method showed that the averages of the two estimates were more accurate than the participants’ first estimates. That is, the wisdom of the inner crowd elicited. In addition, we found that the method could be superior to other methods in terms of efficacy and convenience. Moreover, we identified the conditions where our method worked better. We further clarify the availability and limitations of using the wisdom of the inner crowd. Overall, this paper proposes an effective and short-time method for harvesting the wisdom of the inner crowd.

## Introduction

In daily life, people often need to estimate factual problems (e.g. the number of people who will attend a meeting or the price of a gift), and doing so may be either easy or difficult depending on the particular case. For difficult cases, researchers have sought ways to arrive at accurate estimations. One promising approach is to harness “the wisdom of the crowds”^1–11^: that is, collecting estimations from many people and aggregating them (especially averaging) can yield surprisingly accurate estimates. For over 100 years, various studies have investigated this phenomenon.

**Figure 1:**
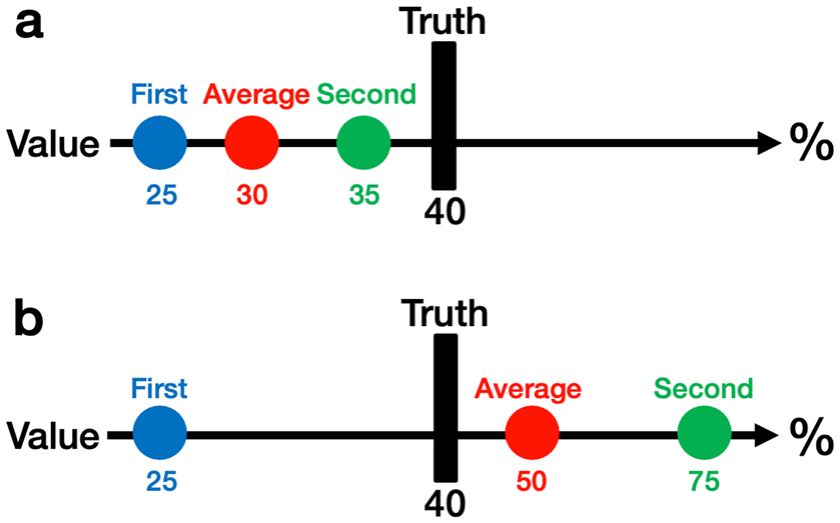
Examples of the wisdom of the inner crowd. (**a**) It emerged when both the first and second estimates were (more than) or less than the true value. (**b**) It emerged when the first and second estimates were on opposite sides with the true value in the middle. Precisely, when the first estimate was 25, the wisdom of the inner crowd emerged if the second estimate was more than 25 and less than 85.

However, the wisdom of the crowds also has a fundamental problem in that it is difficult to gather estimates from many people^12,13^. To solve this problem, previous studies^14,15^ proposed methods that exploited the wisdom of the crowds within-person (hereafter, “the wisdom of the inner crowd”^16–20^). In such approach, an individual is asked to produce two different estimates in response to a single question, in place of two people’s estimates. Research has shown that the average of an individual’s two estimates can be more accurate than their first estimate. For instance, in Fig. 1, the true value response to the question is 40. The participant gave a first estimate of 25 and a second estimate of 35 (Fig. 1a). These average to 30, which is closer to the true value when compared to the first estimate of 25. Figure 1b shows a first estimate of 25 and second of 75, with the true value 40 in the middle. Subsequently, the average is 50, which is better than 25.

For collecting the wisdom of the inner crowd, two promising methods have been proposed thus far. The first is to utilise the power of forgetting^14^. A study^14^ indicated that by providing a timespan between two estimates (e.g. 2 weeks), people can exploit the wisdom of the inner crowd. The second is to utilise the power of the dialectic^15^. For instance, previous studies^15,20^ have shown the effectiveness of making people “consider the opposite” when making second estimates (called Dialectical bootstrapping; see Table 1 for more details).

**Table 1:** Full instructions for each method (called “condition” in our experiments).

Method	Instructions for the second estimates
Other’s perspective	How do you think people in general estimate the following question? Make a second estimate after considering fully how people in general estimate this. (The computer display does not present the first estimate).
Dialectical	First, assume that your first estimate is off the mark. Second, think about a few reasons why that could be. Which assumptions and considerations could have been wrong? Third, what do these new considerations imply? Was the first estimate too high or too low? Fourth, based on this new perspective, make a second, alternative estimate. (The computer display shows the first estimate).
Repeated	No instructions (The display does not present the first estimate).

Note that in second estimates, the Dialectical method required the first estimate to be displayed while the Other’s perspective and repeated methods did not. All instructions were translated into Japanese.

Based on these two methods, many studies over the last decade^16–28^ have examined the wisdom of the inner crowd. However, except for Calseyde & Efendić (2022)’s promising approach^16^, nothing other than the above two methods has been proposed (see the “Discussion” section for more details). As we discuss below, these existing methods leave room for improvements in efficacy and response time. Therefore, this paper proposes a new method that efficiently exploits the wisdom of the inner crowd, which required short time and “boosts”^29–31^ judgments.

For the development of the new method, cognitive and social psychology studies provide important insights. In these fields, many studies have examined how people can think in ways different from their own perspectives. In particular, it is well-known that an individual can access various forms of cognition by considering others’ perspectives, called “perspective-taking”^32,33^. Perspective-taking enables a person to decrease stereotypic biases^34^, change preferential values^35^, and reduce egocentric thinking^36^. Therefore, we primarily decided to follow the perspective-taking paradigm. Subsequently, among others, we considered the general crowd’s viewpoint. People believe that the general crowd is different from themselves in several ways, including degree of intelligence^37–39^ and risk attitude^40,41^. Accordingly, we hypothesized that by considering the general crowd’s perspectives, participants could make estimates different from their own previous estimates in response to the same question.

We developed a new method as follows. Like the two existing methods described above, the method proposed here requires people to give two estimates for a single question. However, unlike the other methods, these procedures ask the participants for their own opinion and then asks them to estimate the opinion of the general crowd or average people (i.e. public opinion; for the full instructions, see Table 1). Subsequently, the two opinions are averaged.

In the sections below, we confirm that the averaged estimates are more accurate than their participants’ original estimates based on their own view. Subsequently, we compare our method with the existing ones in terms of efficacy and convenience, using response time as an index of convenience. The results highlight the room for improvement in the previous methods. Moreover, we identify the conditions in which our method works better (or worse) and point out the limitations involved in utilizing the wisdom of the inner crowd. Through these analyses and discussions, finally, we demonstrate that the new approach can largely aid research on the wisdom of the inner crowd.

## Results

### An overview of the behavioral data

Experiment 1 tested the efficacy of the proposed method, including a comparison with other methods. Therefore, we set three conditions: Other’s perspective, Dialectical, and Repeated conditions. All participants produced two estimates for each question. In the Other’s perspective and Dialectical conditions, participants used our method and dialectical bootstrapping, respectively^15^. Furthermore, in the Repeated condition, they produced two estimates for a question without instructions (Table 1). Participants were randomly assigned to one of the three conditions. The stimulus consisted of the questions which asked general knowledge (for example, “What percent of the world’s airports are in the United States?”; Table 2).

**Table 2:** Questions and correct answers used in the experiments (in %).

Number	Question	Answer
1	The area of the USA is what percent of the area of the Pacific Ocean?	6.32
2	What percent of the world’s population lives in either China, India, or the European Union?	41.29
3	What percent of the world’s airports are in the United States?	32.31
4	What percent of the world’s roads are in India?	7.30
5	What percent of the world’s countries have a higher fertility rate than the United States?	69.84
6	What percent of the world’s telephone lines are in China, USA, or the European Union?	52.09
7	Saudi Arabia consumes what percentage of the oil it produces?	26.62
8	What percentage of the world’s countries have a higher life expectancy than the United States?	22.56
9	What percent of the earth’s surface is covered by water?	70.90
10	What percent of the worldwide land mass is not used for agriculture?	63.10
11	What percent of the world’s population is between 15 and 64 years old?	65.18
12	What percent of the world’s population is Christian?	31.40
13	What percent of the world’s population speaks Mandarin Chinese as a first language?	12.30
14	What percent of the world’s population aged 15 years or older can read and write?	86.30
15	What percent of the worldwide gross domestic product (GDP) comes from the service sector?	63.00
16	What percent of the worldwide labor force works in the agricultural sector?	31.00
17	What percent of the worldwide income does the richest 10% of households earn?	30.20
18	What percent of the worldwide gross domestic product (GDP) is re-invested (“gross fixed investment”)?	25.70
19	What percent of the goods exported worldwide are mineral fuels (including oil, coal, gas, and refined products)?	14.40
20	What percent of the worldwide gross domestic product (GDP) is used for the military (military expenditure)?	2.140

We checked all the answers on 2022/08/05. We used the answers in The world factbook^57^, as with the previous studies^14,15^. Experiment 1 used Questions 1–8^14^, and Experiments 2 and 3 used all the questions^15^. Note that since we could not confirm the answer on Q10, we used the data on the World Bank Data^58^. In addition, as for Q5, we used the latest data on the World Population Review^59^ because fertility rate changes frequently. All questions were translated into Japanese.

In Experiment 2, we also set the three conditions (that is, the Other’s perspective, Dialectical, and Repeated conditions). To conduct further analysis, we performed Experiment 2 with the following modifications from Experiment 1. We recorded response times, asked participants for a third (i.e. final) estimate, and to rate their level of confidence (See more details in “Methods”).

In Experiment 3, we tested the method on an additional framework to examine whether its efficacy increased when the number of estimates increased. In this experiment, we set a single condition: All participants made five estimates for a question, one participant’s own estimate and four estimated public opinions.

### Efficacy of our method

First, we examined whether our method elicited the wisdom of the inner crowd. Therefore, this analysis used the behavioral data from the Other’s perspective condition in Experiments 1 and 2.

**Figure 2:**
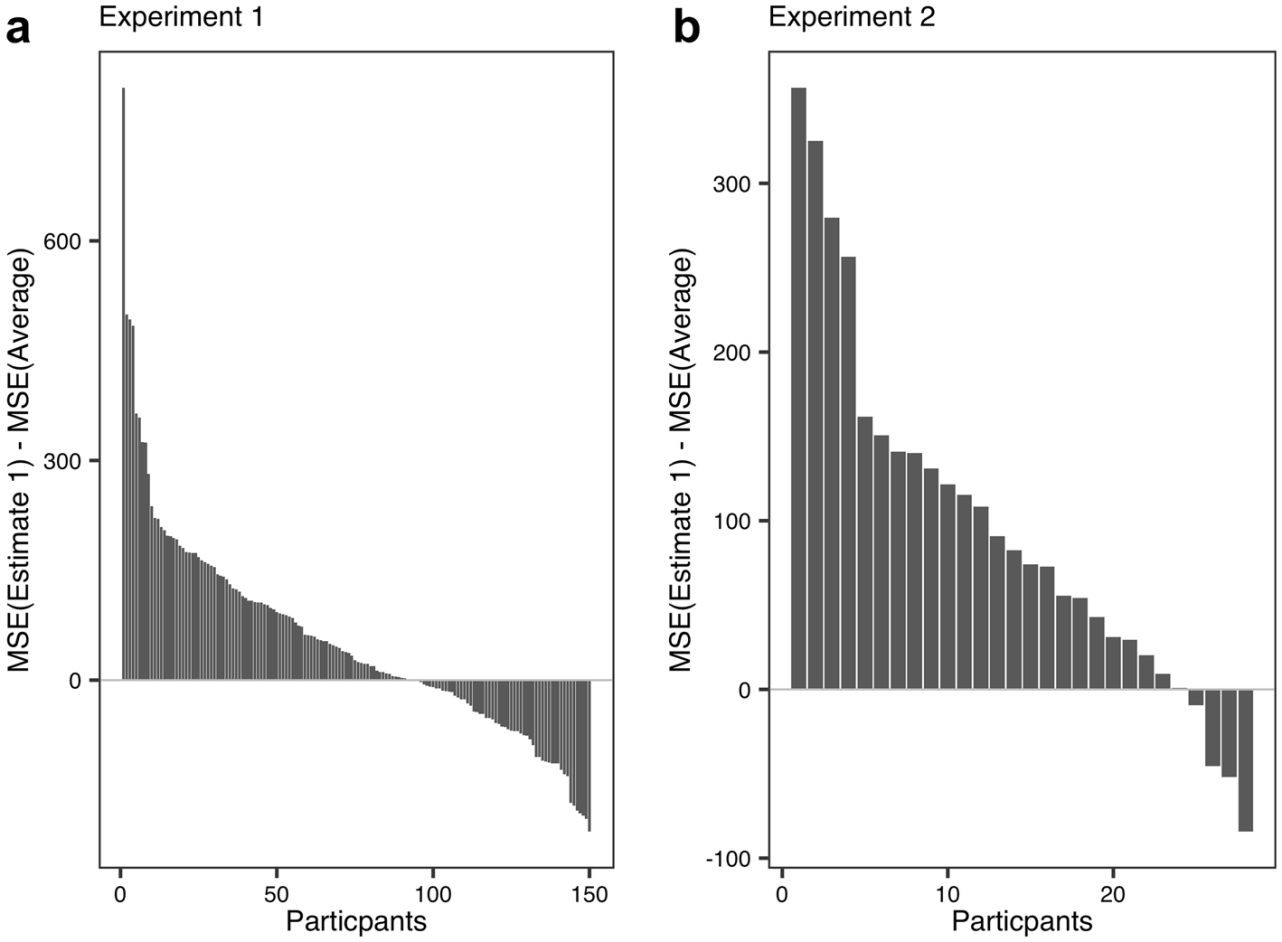
Results for the Other’s perspective condition (our method) in Experiment 1 (**a**) and 2 (**b**). Each bar plot indicates each participant.

Figure 2 shows the results of the analysis. As with the previous study, we conducted calculations for each participant. Especially, we subtracted the MSE in averaged estimate from the MSE in Estimate 1 across all questions. That is, the larger the value, the more accurate is our method.

As a result, in Experiment 1, 93 out of 150 participants recorded positive values (62.00%; *χ*^2^_*1*_ = 9.07, *p* = 0.0026), and in Experiment 2, 24 out of 28 participants recorded positive values (85.71%; *χ*^2^_*1*_ = 14.28, *p* = 0.00016). Thus, we confirmed that the new method elicited the wisdom of the inner crowd.

Note that we also compared the averaged estimates with two people’s own estimates (see Section S1 of the Supplementary Information) and found they did not exceed the two people’s estimates.

### Comparison of the methods

We compared the efficacy among the conditions, based on the data from Experiments 1 and 2. The reduction of the MSE was calculated, as shown in Eq. (1):

1 $$Reduction \,\,of\,\, MSE = {MSE}_{first \,\,estimates} - {MSE}_{averaged \,\, two \,\, estimates}.$$

**Figure 3:**
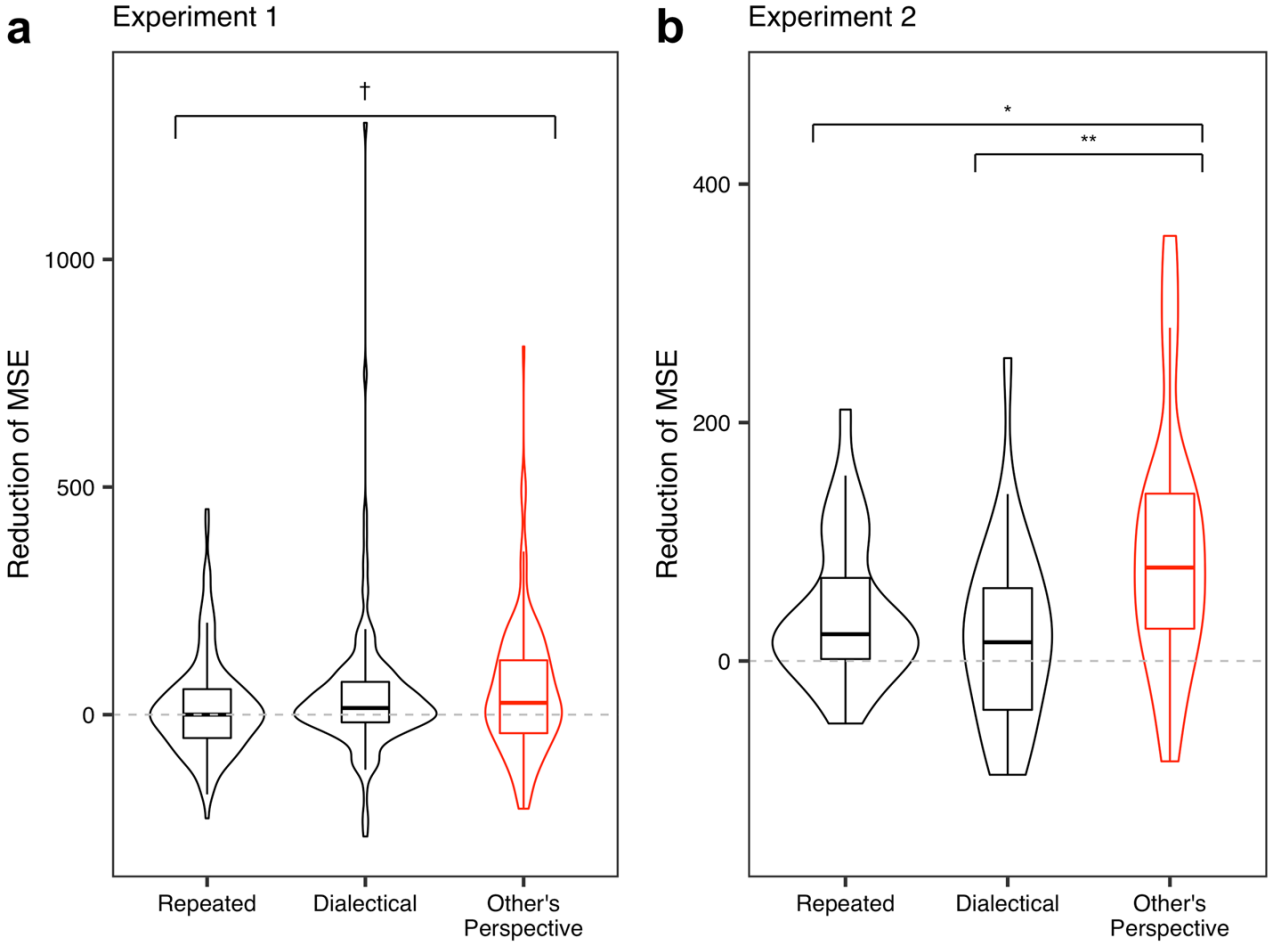
Results for all conditions. 95% CI in Experiment 1, Other’s perspective = [29.40, 75.63], Dialectical = [19.69, 68.92], Repeated = [− 1.46, 33.70] in Experiment 2, Other’s perspective = [57.66, 136.18], Dialectical = [− 5.57, 51.47], Repeated = [20.00, 63.72].

Subsequently, a higher reduction in the MSE indicates a higher effectiveness of a method. As Fig. 3 shows, the results indicate the advantage of our method. We also conducted a mixed-effects analysis^42^, with the reduction of the MSE, condition, and participants and questions as the dependent, independent, and random variables, respectively (see “Mixed-effect analyses” in the “Methods” section).

In Experiment 1, although the significant effect was marginal, the Other’s perspective condition had a larger reduction in the MSE than the Repeated condition (*t*_*449*_ = 2.28, *p* = 0.059). Importantly, in Experiment 2, the Other’s perspective condition had a larger reduction in the MSE than both the Dialectical and Repeated conditions (Dialectical: *t*_*82*_ = 3.23, *p* = 0.0050; Repeated: *t*_*82*_ = 2.46, *p* = 0.042).

Although no significant effects were found between the Other’s perspective and Dialectical condition in Experiment 1 (Dialectical:* t*_*449*_ = 0.62, *p* = 0.81), the results implied the superiority of our method. We also conducted a meta-analysis that combined the data of Experiments 1 and 2 (see Section S2 of the Supplementary Information). We found that, although marginally, the Other’s perspective condition had a larger reduction in the MSE than the Dialectical condition.

### Analysis of the response times

Since methods for eliciting the wisdom of the inner crowd can be used on a daily basis, it is important that they are convenient to use. Thus, we compared the response times among all of the conditions.

In Experiment 2, the laboratory computer recorded the response times. Particularly, we examined the response times for the second estimates because, for these estimates, the participants were instructed differently, depending on their assigned condition (Table 1).

**Figure 4:**
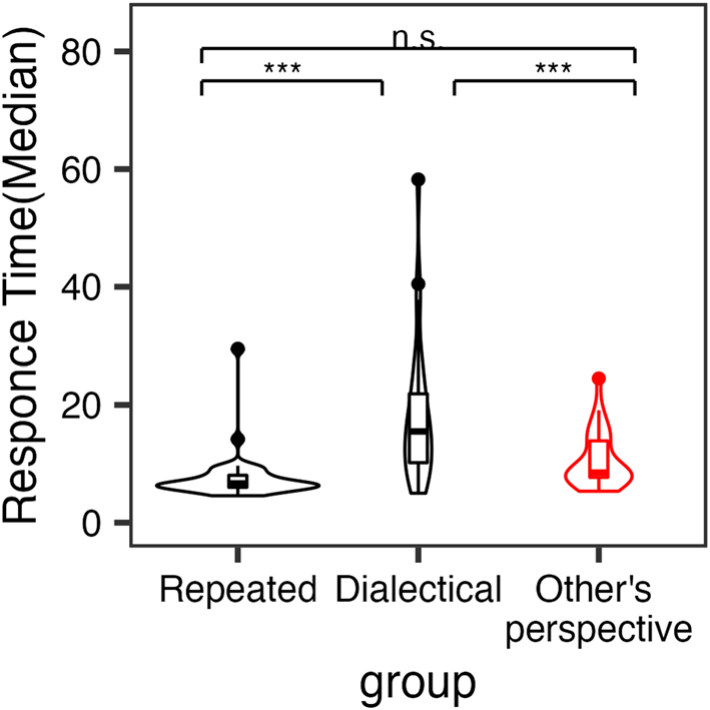
Results of the response time among three conditions in Set 2 of Experiment 2. 95% CI Other’s perspective = [8.94, 12.28], Dialectical = [14.04, 22.99], Repeated = [6.58, 9.58].

Figure 4 shows the results of the analysis. Specifically, we conducted a mixed-effects analysis^42^, with response time as the dependent variable, condition as the independent variable, and the participants and questions as the random variables.

Notably, the Other’s perspective condition had a significantly shorter response time than the Dialectical condition (*t*_*82*_ = 4.00, *p* = 0.00040). Given that a method that requires the participants to spend more time is possibly overloaded, the results imply that our method might have a lower cognitive load than that of the previous study.

Meanwhile, the repeated condition had a shorter response time than the Dialectical condition (*t*_*82*_ = 5.78, *p* < 0.0001). However, we did not find any significant effects between the Repeated and Other’s perspective condition (*t*_*82*_ = 1.72, *p* = 0.20). Although our method included instructions, the participants did not spend more time to respond, compared to the condition in which they did not receive specific instructions. Hence, along with the results in the last section, our method is superior to other methods in terms of efficacy and response times.

### When the proposed method worked better (or worse)

For further analysis, we investigated the conditions under which the methods worked better or worse. In Experiment 2, all participants reported their level of confidence in their first estimates. Subsequently, we analyzed the influence of confidence on the efficacy of each method. We conducted mixed-effects analyses^42^ for each condition with the reduction of the MSE, confidence intervals, and participants and question as dependent, independent, and random variables, respectively.

**Figure 5:**
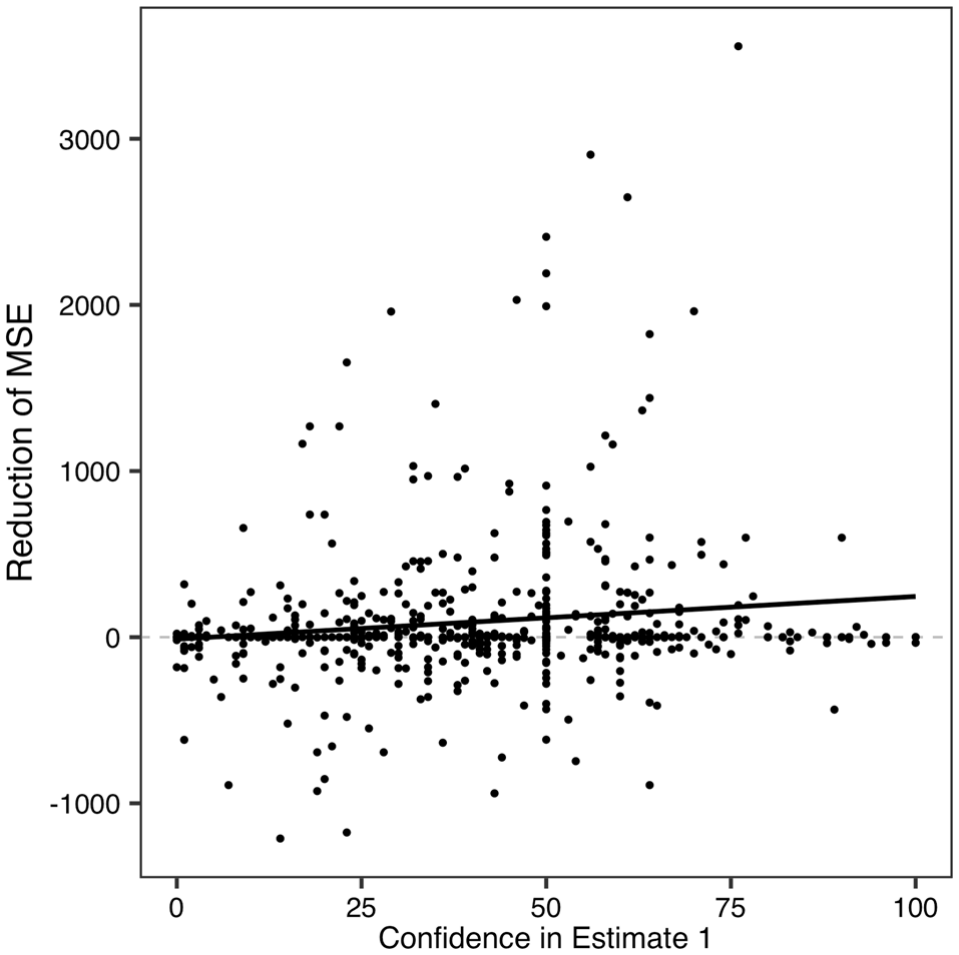
Results of the relationship between confidence in the first estimate and the reduction of MSE in the Other’s perspective condition in Experiment 2. The black line represents the regression line.

The results showed that in the Other’s perspective condition, higher confidence corresponded to a greater reduction of MSE (*F*(1, 531.62) = 10.30,* p* = 0.0014; see also Fig. 5). In other words, the proposed method worked better when participants were confident in their own estimates. Accordingly, it would be better for people to use the method when their confidence is high. For other conditions, we did not find such effects (Dialectical condition: *p* = 0.96; Repeated condition: *p* = 0.73). Thus, hereafter, we shall discuss the Other’s perspective condition.

How did these results emerge? In Experiment 2, confidence did not correlate with accuracy in the first estimate (*p* = 0.29), meaning there is room for improving estimates even when the participants feel confident. Importantly, confidence in the first estimate correlated with accuracy in the second estimate. We conducted an additional mixed-effects analysis that included the MSE in the second estimate as a dependent variable, with confidence as an independent variable. The results showed that, although marginally, the higher the confidence was, the lower the MSE was in the second estimate (*F*(1, 544.9) = 2.80, *p* = 0.095). Subsequently, the average could be close to the true value, resulting in the consequences as described above. We shall remark on this issue in the “Discussion” section.

### The accuracy of the final estimates and the change in confidence

Previous studies^19,20^ have discussed the possibility that people cannot utilise the wisdom of the inner crowd. As mentioned earlier, the average of the two estimates in the proposed method was accurate for harvesting the wisdom of the inner crowd. However, people might not naturally use averages as their final estimates. For example, some people might adopt their first estimate as their final one. Hence, we addressed this problem, based on the results of Experiment 2, since all of the participants produced final answers based on their own thinking.

**Figure 6:**
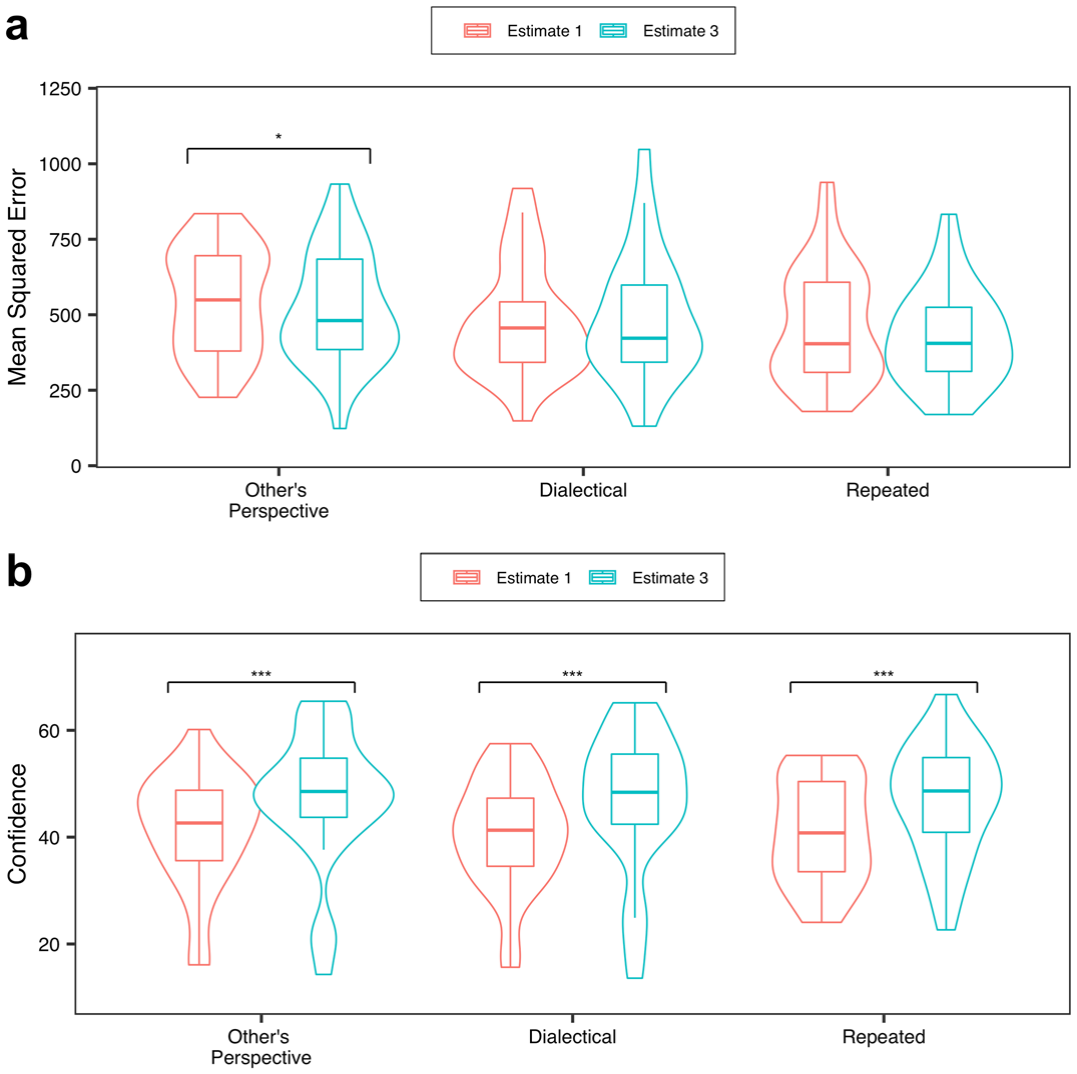
Results of the final estimate as for (**a**) Mean Squared Error and (**b**) confidence. (**a**) Only in the other’s perspective condition, the final estimate was more accurate than the first estimate (*p* = 0.028). 95% CI Other’s perspective in Estimate 1 = [482.01, 609.87], in Estimate 3 = [453.02, 588.27]; Dialectical in Estimate 1 = [406.88, 540.03], in Estimate 3 = [406.59, 559.19]; Repeated in Estimate 1 = [409.01, 547.49], in Estimate 3 = [359.65, 471.49]. (**b**) In all conditions, people became more confident in the final answer (*ps* < 0.0001). Note that between the conditions, no significant differences were found in either Estimate 1 or Estimate 3 (*ps* = 1.00). 95% CI Other’s perspective in Estimate 1 = [37.53, 45.40], in Estimate 3 = [42.65, 51.64]; Dialectical in Estimate 1 = [36.72, 44.27], in Estimate 3 = [41.83, 51.51]; Repeated in Estimate 1 = [37.66, 44.43], in Estimate 3 = [43.06, 50.52].

Figure 6a presents the results of the analysis, which compared the MSEs of the first and final estimates. We also conducted mixed-effects analyses^42^ for each condition, with the MSE, Estimate (i.e. Estimates 1 or 3), and participants and questions as the dependent, independent, and random variables, respectively.

As Fig. 6a shows, only in the Other’s perspective condition, the final estimate was more accurate than the first estimate (*t*_*1072*_ = 2.20, *p* = 0.028). Thus, in contrast to the findings of previous studies, our method allows participants to naturally elicit the wisdom of the inner crowd. Meanwhile, in the Repeated and Dialectical conditions, we did not find such effects (Repeated: *t*_*1150*_ = 1.12, *p* = 0.26; Dialectical: *t*_*1033*_ = 0.57, *p* = 0.57). In these methods, people do not naturally utilise the inner crowd (for more details, see Section S3 of the Supplementary Information).

Figure 6b includes the findings of the additional analysis. Specifically, we conducted a mixed-effects analysis^42^ for each condition, with Confidence (i.e., Confidence in Estimate 1 or Confidence in Estimate 3), Estimate, and participants and questions as the dependent, independent variable, and random variables, respectively.

In Experiment 2, the participants responded with confidence in their final estimates. Compared to their confidence in their first estimates, we found that the participants became more confident in their final estimates across all conditions (Fig. 6b, Other’s perspective:* t*_1072_ = 6.07, *p* < 0.0001; Dialectical: *t*_1033_ = 6.30, *p* < 0.0001; Repeated: *t*_1040_ = 5.69, *p* < 0.0001). As mentioned earlier, in the Other’s perspective condition, the participants were able to elicit the wisdom of the inner crowd. However, in the Dialectical and Repeated conditions, despite the fact that the participants did not elicit the wisdom of the inner crowd, they became more confident^43,44^ (Note the so-called “overconfidence”^45–48^; see the previous definition).

### When the number of estimates increased

At this point, we had asked the participants to provide a single public opinion in the Other’s perspective condition. Subsequently, can the efficacy of our method increase if the number of estimated public opinions increases? Previous research on the wisdom of the inner crowd^18,19,23^ has discussed the effect of the number of estimates. For instance, one study^23^ examined a case in which the participants gave five estimates in response to a single question (note that this study provided no specific instructions). Then, they compared this case with the condition in which the participants gave two estimates for a question. Based on the findings, increasing the number of estimates did not enhance the wisdom of the inner crowd effect. Thus, to determine the potential of our method, it is important to determine whether the number of estimates can enhance the wisdom of the inner crowd effect.

In Experiment 3, all of the participants gave five estimates in response to each question, i.e. the participants answered their own estimates once and estimated public opinions four times (for more details, see the “Methods” section). In the analysis, we calculated the reduction of the MSE. In this context, we computed how much the error in the first estimate decreased by averaging all five estimates. Hence, the reduction of the MSE was calculated, as shown in Eq. (2):

2 $$Reduction\,\, of \,\,MSE = {MSE}_{first\,\, estimates} - {MSE}_{averaged\,\, five\,\, estimates}.$$

**Figure 7:**
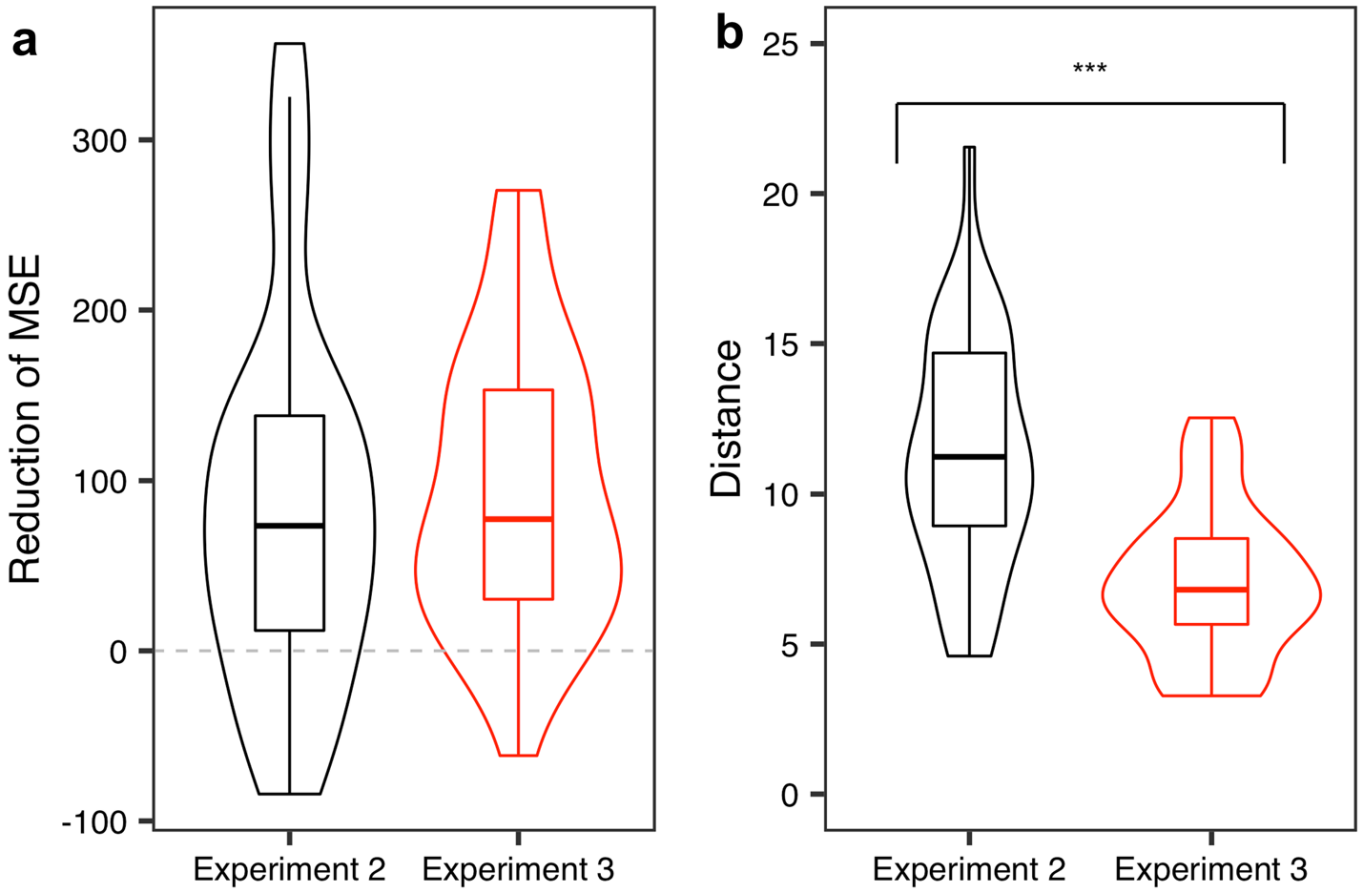
Results of the analysis for Experiment 3. (**a**) compares Experiments 2 and 3 in terms of the reduction of MSE. 95% CI Experiment 2 = [57.20, 134.92]; Experiment 3 = [65.65, 123.38]. (**b**) compares the distance between Experiments 2 and 3. 95% CI Experiment 2 = [9.72, 12.57]; Experiment 3 = [6.17, 7.95].

Subsequently, we compared the results of this analysis with those of the Other’s perspective condition in Experiment 2. As Fig. 7a shows, the reduction of the MSE in Experiment 3 showed a positive value (95% CI  47.20, 126.91; we conducted bootstrapping based on 10,000 sampling with replacement). In other words, the error in the first estimate decreased to some degree when the participants presented five estimates.

However, notably, we could not find any significant effects between them (*t*_*57*_ = 0.032,* p* = 0.97). We also conducted a mixed-effects analysis^42^, with the reduction of the MSE, Experiment (i.e. Experiment 2 or Experiment 3), and participants and questions as the dependent, independent, and random variables, respectively.

This indicates that increasing the number of public opinion estimates did not necessarily enhance the efficacy of our method (see the “Discussion” section for speculation on how to overcome this limitation).

How did the results emerge? As mentioned in the Introduction, the wisdom of the inner crowd paradigm aims to make participants produce different opinions from their own. Subsequently, we calculated Distance in both experiments. Here Distance represents the absolute distance between participants’ own and estimated public opinions. As for Experiment 3, we first averaged the four public opinions and computed the distance. We also conducted a mixed-effects analysis^42^ with Distance, Experiment (i.e. Experiment 2 or Experiment 3), and participants and questions as the dependent, independent, and random variables, respectively.

Based on the results, Experiment 3 had a smaller distance than Experiment 2 (see Fig. 7b; *t*_57_ = 4.74, *p* < 0.0001). In other words, Experiment 3 failed to make the participants produce different opinions, compared to Experiment 2.

## Discussion

This study proposes a new method exploiting the wisdom of the inner crowd. Our method asks participants to give two estimates in response to a question: their own estimate and their estimate of public opinion. It then averages the estimates (as for optimal weighting, see S7 for Supplementary Information). Across Experiments 1 and 2, we confirmed that the proposed method produced the wisdom of the inner crowd effect. Moreover, we found that it could be more effective and convenient than other methods.

Moreover, this study makes two substantial contributions. First, we identified the conditions under which the new method works better or worse. These conditions have been given little attention in the existing literature, to the best of our knowledge. However, it is important to understand efficacy in context to appropriately utilise a method for eliciting the wisdom of the inner crowd for informing decisions encountered in daily life. The analysis showed that our method worked better when participants had high confidence in their own estimates.

As mentioned above, we found that the accuracy of the second estimate was high when the confidence in the first estimate was high. We speculated the cause of the results as follows. When participants had low confidence in their response, it might be difficult for them to estimate public opinion, resulting in producing estimates that was off the mark (e.g. the answer was 10 and an estimate was 90). In contrast, when they had a higher degree of confidence, it seemed to be relatively easy to produce a plausible estimate of public opinion. For future study, we plan to examine this speculation directly: put simply, when participants estimate public opinions, we will ask them how confident they feel in taking different perspectives.

Furthermore, this study implied that participants could naturally use our method. Especially, in Experiment 2, we made participants produce their final answers; their final estimates were more accurate than their first estimate. This also indicates the superiority of our method since the results did not emerge in other methods.

In the additional analysis, we found that they became more confident about the third or final estimate compared to the first estimate. However, other than our method, accuracy did not increase. We assume that this could be a defect of methods that elicit the wisdom of the inner crowd.

The results also point to the limitations of utilizing the wisdom of the inner crowd. Although the number of estimates (that is, simulated public opinions) increased, the efficacy of our method did not increase. However, a previous study^23^ also showed that theoretically, the efficacy of the methods could increase as the number of estimates increased. Thus, we consider our results as showing the need for a method that is better structured.

Another limitation of this study is the types of questions used in the experiments. Based on previous studies, we used questions that included the percentage of correct answers. However, there are other types of questions (e.g. years of historical events and numerical estimation tasks). The previous study reported that concerning the wisdom of inner crowds, the effects were basically maintained across different types of questions. However, it remains unclear whether our method also elicited the wisdom of inner crowds in other types of questions. Thus, further research, which aims to generalize this study’s findings for the types of questions, is necessary.

Overall, as for both the potentials and limitations, this paper highlights room for further analysis of methods on the wisdom of the inner crowd. Note that previous research can be classified into two directions. First, some studies have attempted to extend the wisdom of the inner crowd to other tasks^24–27,35^. For example, a previous study^27^ showed that an individual could improve performance evaluations of matters for which there is no objective truth (for other examples, see social projection^25^ and self-deception^26^) by using this method. Second, other studies^22–24^ focused on its theoretical mechanism. For example, a previous study^23^ presented a mathematical framework indicating two mechanisms through which the wisdom of the inner crowd works.

In contrast, the method itself has been little proposed. This may be derived from the difficulty of developing the methods^15^. The second estimate should differ from the first estimate; however, at the same time, the second estimate should be a plausible one. If the second estimate only adds noise^49,50^, the average estimate will not be more accurate than the first one. Nevertheless, this study demonstrates that developing an alternative method to those already proposed can facilitate research on the wisdom of the inner crowd.

As mentioned in the Introduction, a new promising method^16^ that exploits the wisdom of the inner crowd has recently been proposed. This method requires participants to produce two estimates for a single question. Specifically, they first answer their own estimate and then create another estimate from the perspective of someone with whom they disagree (hereafter called “disagree-other”). Thus, we obtain four methods that exploit the wisdom of the inner crowd: (1) providing a timespan^14^; (2) dialectical bootstrapping^15^; (3) disagree-other^16^; and our method (Strictly, Winkler, and Clemen^51^ also suggested another intervention type).

There are similarities between the disagree-other method and our method. First, both methods utilise the perspective-taking paradigm^32,33^. Second, both methods assume that simply taking another person’s point of view is insufficient for producing different estimates from an individual’s own perspective. Meanwhile, there are several differences between the two methods. First, the disagree-other method^16^ makes participants take an individual’s perspective, while our method makes participants simulate the crowd’s perspective. Second, the disagree-other method makes participants take someone’s perspective, who probably has a different opinion from their own estimates. In this regard, we can assume that the former method is similar to dialectical bootstrapping. In contrast, our method does not require participants to re-consider their own estimates, at least explicitly.

In sum, besides the providing a timespan method^14^, the dialectical bootstrapping^15^, the disagree-other^16^, and our method have similarities and differences. These differences can play a role in exploiting the wisdom of the inner crowd, as shown in the “When the proposed method worked better (or worse)” section. However, more research that compares these methods should be conducted.

This study is also related to previous studies, especially regarding the social circle by Galesic, Bruine de Bruin, and others^52–55^. These studies have shown that by using an individual’s knowledge of their social circle, we can improve predictive performance such as political poles^52^. We can also assume that a response based on the knowledge of the social circle is similar to the estimate from the crowd’s perspective.

However, the objective for producing different responses and estimates from their own perspective is different. In contrast to previous research, the estimate from the crowd’s perspective itself is not necessarily accurate for our method. In other words, the aim of our method is to obtain an accurate average estimate.

Finally, we can connect this study with the pivoting method proposed by Palley and Soll^56^. Although the aim of this particular method is to maintain the accuracy of the crowd’s estimate and not exploit the wisdom of the inner crowd, the elicitation is quite similar. For example, in their Study 3, for numerical judgment, the participants first created their own estimates and then guessed the average estimate of all of the other participants.

It should be added that the purpose of the pivoting method is to improve the shared-information problem. As the wisdom of the crowd effect showed, the independent estimates of the crowd were quite accurate. However, in practice, information was often shared among the crowd. As a result, the estimates were correlated, which decreased the wisdom of the crowd effect.

In this regard, we may consider our method (and all methods that exploit the wisdom of the inner crowd) as addressing the shared-information problem’s within-person level. Moreover, without a timespan and any specific instructions, an individual is supposed to produce two similar estimates, since the same individual has shared information. Therefore, to access other information and avoid the shared-information problem, our method makes participants produce estimates from the crowd’s perspective.

## Methods

In all three experiments, the participants provided informed consent prior to joining the study. The experimental protocol was approved by the University of Tokyo Research Ethics Committee and conducted in accordance with the latest version of the Declaration of Helsinki.

### Experiment 1: participants and procedure

The participants were 452 Japanese adults who participated in the experiment through a web research company. To gather the participants in the web-based survey, we contracted with Rakuten Insight (https://member.insight.rakuten.co.jp/), a well-known investigation company. Rakuten Insight has the largest panel in Japan, consisting of more than 220,000 people. After completing the study, the participants received cash-equivalent points as an incentive that could be used for online shopping.

The stimuli were eight questions about general knowledge (e.g. “What percent of the world’s airports are in the United States?”; Table 2), the same as in the previous study^14^. The participants answered the question set twice (Sets 1 and 2), yielding a total of two estimates in response to each question. Across the two sets, the order of questions remained constant. Further, we randomized the order of questions for each participant.

There were three conditions: Other’s perspective, Dialectical, and Repeated. The participants were randomly assigned to one of the three conditions (Other’s perspective: *n* = 150, 98 female and 52 male, *M*_*age*_ = 45.5, and *SD*_*age*_ = 8.0; Dialectical: *n* = 151, 95 female and 56 male, *M*_*age*_ = 43.6, and *SD*_*age*_ = 8.0; Repeated: *n* = 151, 94 female and 57 male, *M*_*age*_ = 43.6, and *SD*_*age*_ = 8.1). In the Other’s perspective condition, they used our method of answering Set 1 with their own opinion and Set 2 with their estimate of public opinion. In the Dialectical condition, they did dialectical bootstrapping^15^, and in the Repeated condition, they answered both sets without instructions on what to think about (Table 1).

### Experiment 2: participants and procedure

The participants were 90 Japanese undergraduate and graduate students. They received a flat fee of 1000 Japanese yen (approximately $9.17 at the currency rate at the time) for their participation.

The stimulus consisted of 20 questions about general knowledge (Table 2), the same as in the previous study^19^. The participants answered the question set three times (Sets 1–3), yielding a total of three estimates in response to each question. Across the three sets, the order of questions remained constant. Furthermore, we randomized the order of questions for each participant.

As in Experiment 1, the participants were randomly assigned to one of the three conditions (Other’s perspective: *n* = 30, 7 female and 21 male, *M*_*age*_ = 21.2, and *SD*_*age*_ = 2.3; Dialectical: *n* = 29, 8 female and 20 male, *M*_*age*_ = 21.1, and *SD*_*age*_ = 3.1; Repeated: *n* = 31, 8 female and 23 male, *M*_*age*_ = 20.6, and *SD*_*age*_ = 2.5). We dropped the data of three participants based on demographic data (two in the Other’s perspective condition and one in the Dialectical condition).

We were unable to measure the confidence of the first five participants (two in the Other’s perspective condition, two in the Dialectical condition, and one in the Repeated condition). Thus, we excluded this data from all of the analyses.

In Set 1, they gave their own estimates across all conditions. They also evaluated their level of confidence in each response on a scale ranging from 0 (I am not confident in my answer at all) to 100 (I am very confident in my answer). In Set 2, they gave estimates depending on their assigned condition, as in Experiment 1. Subsequently, in Set 3, they were shown their two previous estimates on the computer display and asked to provide a final answer along with their level of confidence in it.

### Experiment 3: participants and procedure

The participants were 33 Japanese undergraduate and graduate students (14 female and 19 male, *M*_*age*_ = 20.0 and *SD*_*age*_ = 1.4). They received a flat fee of 1000 Japanese yen (approximately $9.17 at the currency rate at the time) for their participation.

The stimulus consisted of the same 20 questions used in Experiment 2. We set only one condition in this experiment: All the participants gave five estimates for each question (Sets 1–5). First, they answered with their own estimate, and then they gave estimates of public opinion four times all at once.

### Mixed-effect analysis

We performed all mixed-effects analyses using the R packages *lme4* and *lmerTest*^42^. We selected the best model and computed all statistical values using the *step()* function for the full model with random participants and stimulus intercepts. We performed all multiple comparison using the R packages, *lsmeans* and *pbkrtest*.

## Supplementary Information

Supplementary Information.

## Data Availability

The R-code and the three datasets analyzed in this study are available in the Mendeley Data: https://data.mendeley.com/datasets/p29rkjmvjp/1.
