# Supplementary material for: On an effective and efficient method for exploiting the wisdom of the inner crowd
Source: Sci Rep. 2023 Mar 3;13:3608. doi: 10.1038/s41598-023-30599-8 (PMC9984468; doi:10.1038/s41598-023-30599-8)
Supplement: Supplementary file 1 — Supplementary Information. [file 41598_2023_30599_MOESM1_ESM.docx]

Supplementary materials

On an effective and efficient method for exploiting the wisdom of the inner crowd

*Itsuki Fujisaki^1^, Kunhao Yang^2^, & *Kazuhiro Ueda^1^

^1^ Graduate School of Arts and Sciences, The University of Tokyo; Tokyo, Japan

^2^ Faculty of Law, Chuo Gakuin University; Osaka, Japan

*e-mail: bpmx3ngj@gmail.com (I.F.), ueda@g.ecc.u-tokyo.ac.jp (K.U.)

**This PDF file includes:**

Supplementary text

Figures S1 to S13

Tables S1 to S6

**S1****. Compared with two people in the Other’s perspective condition**


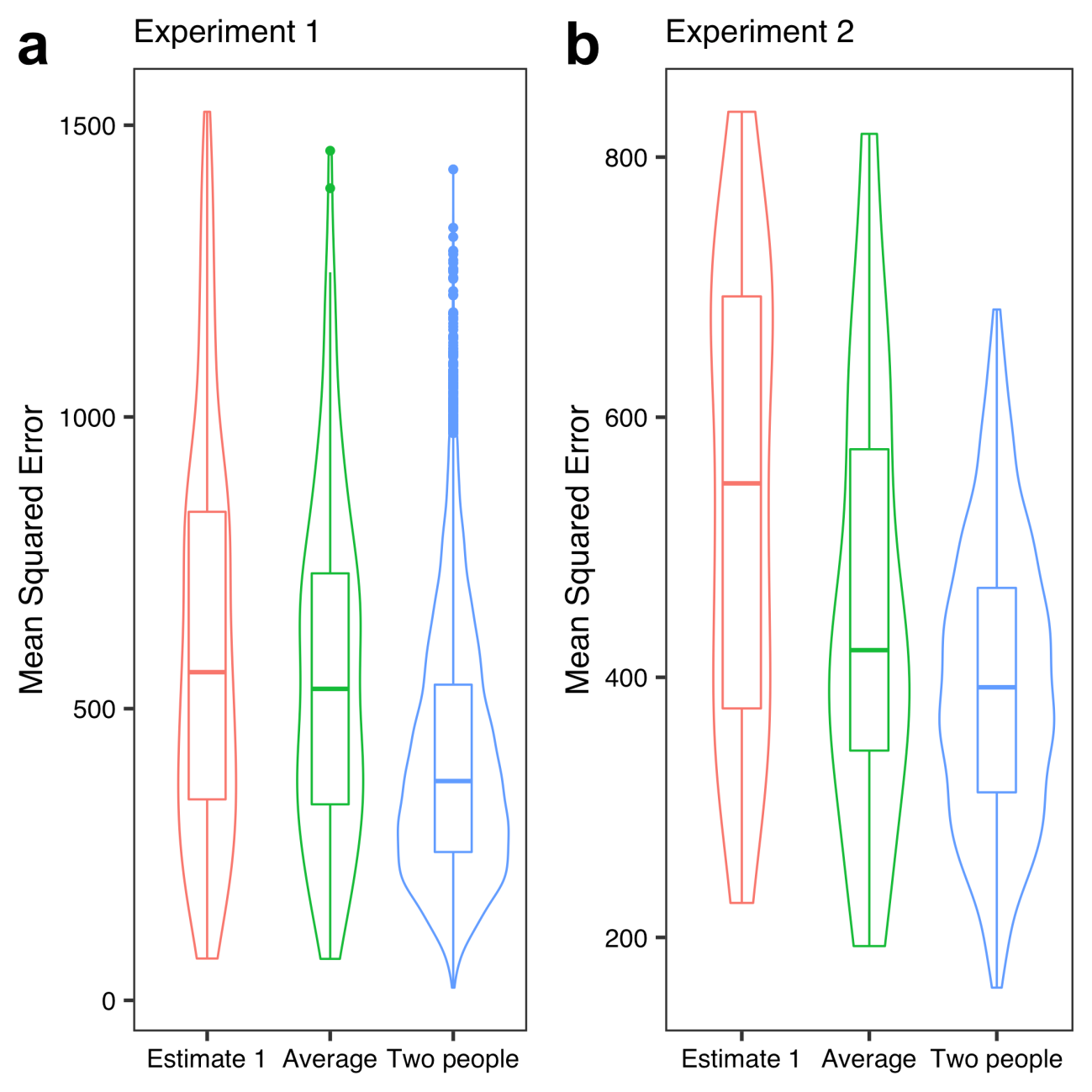


**Fig. S1. Results of the analysis in Experiment 1(a) and Experiment 2(b). For the “Average” category, Estimate 1 and Estimate 2 were averaged. For the ”Two people” category, all combinations of two people were selected, and their separate Estimate 1 responses were averaged. Subsequently, the MSE was calculated. As this figure shows, our method could not beat two people’s estimates.**

**Table S1. Average MSE for each estimate in Experiments 1 and 2.**

|  | Estimate 1 | Average | Two people |
| --- | --- | --- | --- |
| Experiment 1 | 615.82 | 564.02 | 414.39 |
| Experiment 2 | 541.09 | 455.53 | 396.99 |

**S2. Meta-analysis regarding the comparison of the methods**

In this section, we conducted a meta-analysis that combined the data of Experiments 1 and 2. Specifically, we first calculated the reduction of the MSE across 8 (Experiment 1) and 20 questions (Experiment 2) for each participant, respectively, after which we combined the data. We also conducted a mixed-effects analysis, with the reduction of the MSE, condition, and participants and questions as the dependent, independent, and random variables, respectively.

As Fig. S2 shows, the Other’s perspective condition had a larger reduction of the MSE than the Repeated condition (*t_465_* = 3.18, *p* = .0045). Importantly, we also found a marginally significant effect between the Other’s perspective and the Dialectical condition (*t_474_* = 2.13, *p* = .084). **
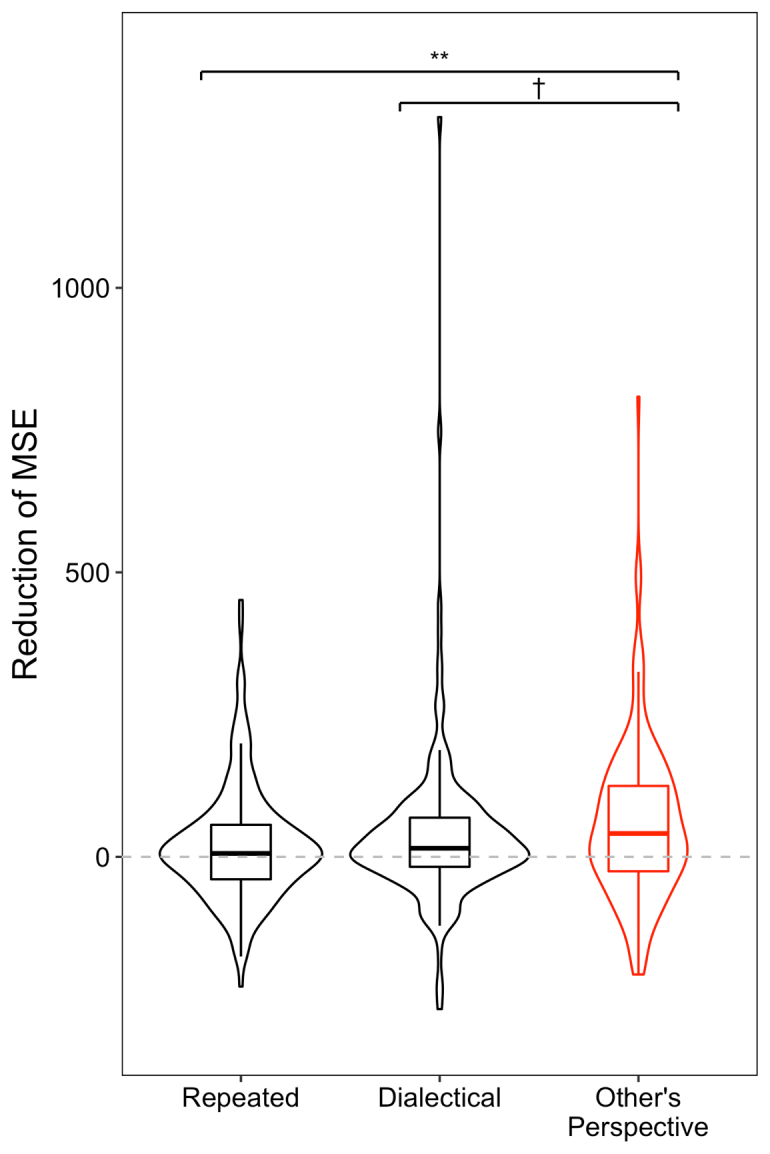
**These results suggest that our method might be better than dialectical bootstrapping.

**S3. Data on final estimates**

The findings revealed that the third (i.e., final) estimates were not more accurate than the first estimates, except for in the Other’s perspective condition. To investigate why, we conducted an additional analysis on the final estimates. We calculated how much the final estimates were weighted on Estimate 2, using the following equation.

$$Weighting on Estimate 2 = 1-((Estimate 3 - Estimate 1) / (Estimate 2 - Estimate 1))$$

Fig. S3 shows the results of the analysis. There were many cases where the values of weighting on Estimate 2 were zero and one. In these cases, participants could not harness the wisdom of the inner crowd effect.


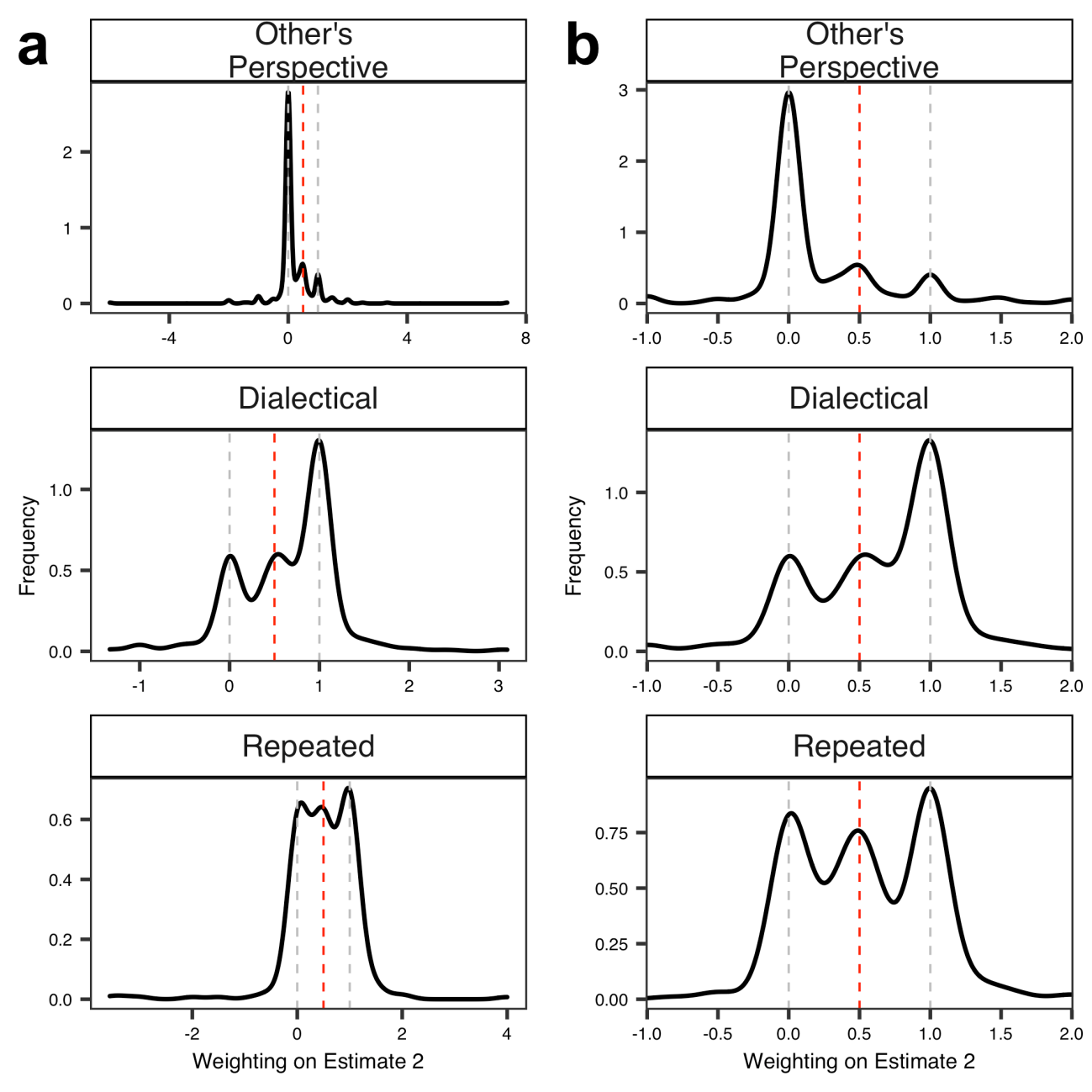


**Fig. S3. Results of the analysis of final estimates(a). Each black line represents a probability density function. In (b), for simplicity, we show the case where the value of the weighting on Estimate 2 was from -1.0 to 2.0. The dotted red line indicates the case where the third estimate was the average of Estimate 1 and Estimate 2. The dotted gray lines represent the case where the final estimate was the same as Estimate 1 or Estimate 2. Note that when Estimate 1 and Estimate 2 had the same value, we could not calculate the weighting on Estimate 2. Therefore, we excluded these cases from the analysis.**

**S4. Analysis of the relationship between confidence in Estimate 1 and the final estimate**

In Experiment 2, the participants rated their level of confidence in their own estimates and gave a final answer. What is the relationship between them? One might think that less-confident participants put more weight on their public opinions. However, we did not find such results.

Specifically, we examined the relationship between the level of confidence in Estimate 1 and the value of weighting on Estimate 2. For each participant, we computed the mean of these values for all questions and then calculated their correlation (Fig. S4). We found no significant correlations in all conditions.


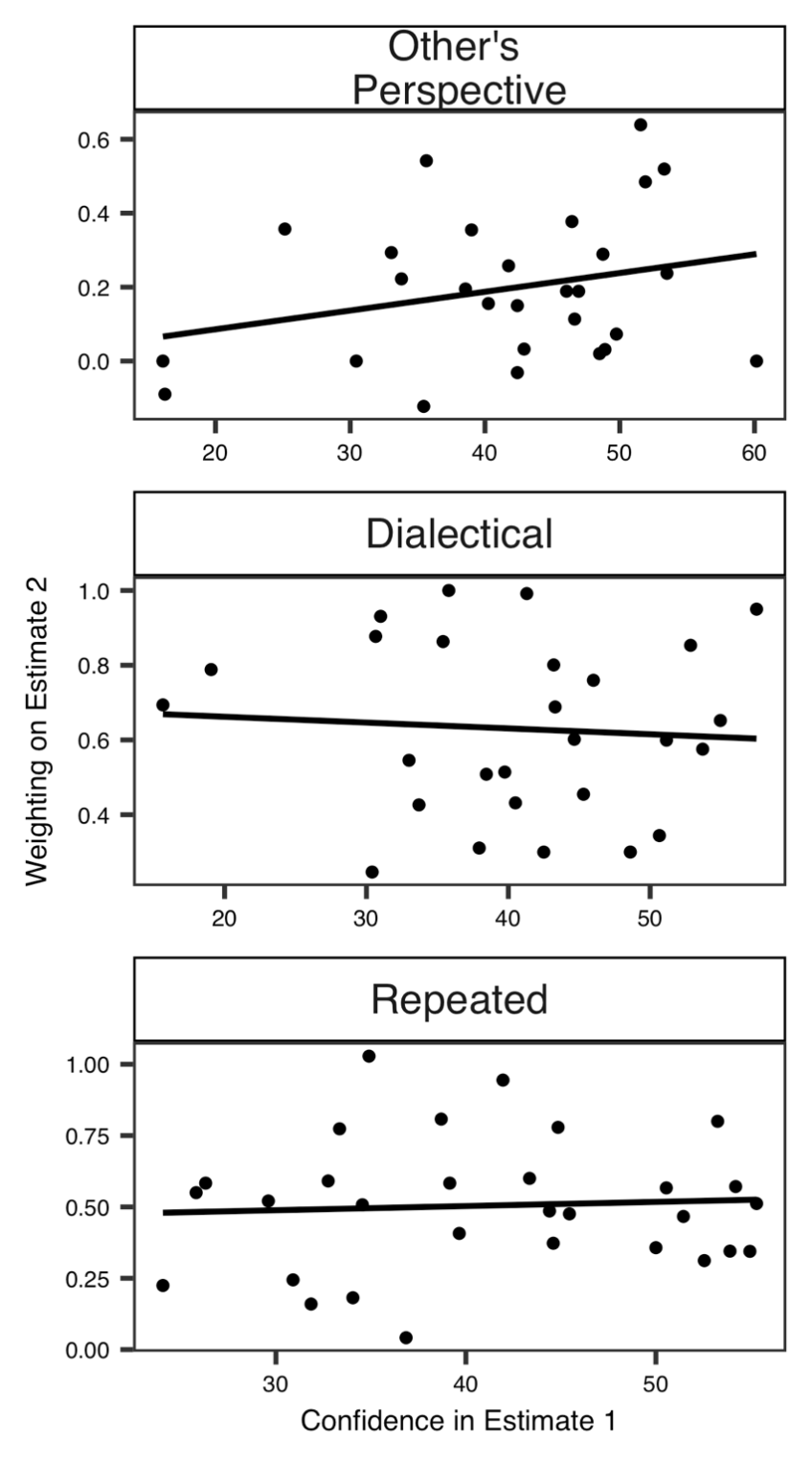


**Fig. S4. Results of the analysis of the relationship between participants’ level of confidence in Estimate 1 and the value of weighting on Estimate 2. Each plot indicates each participant, and black lines represent the regression lines. We found no significant correlations in all conditions (Other’s perspective: *p* = .16; Dialectical: *p* = .73; Repeated: *p* = .75).**

**S5. Additional explanation as for “When the number of the estimates increased”**

Table S2 presents the results of Experiment 3. In this table, we categorised the participants’ own estimates. Specifically, we categorised them according to the relative size compared to five public opinions: the smallest, second smallest, medium, second largest, and the largest. We first counted the number of estimates falling into each category for each participant and then totaled the number for all of the participants. Consequently, the frequencies of appearance of the “medium” and “second smallest” categories were larger than those of the other categories (95% CI).

Especially in the medium category, we can assume that the participants assigned two out of the four public opinions to larger values than their own estimates and assigned the other two opinions to smaller values than their own estimates. In other words, we can consider that the participants’ initial estimates functioned like anchoring.^1–3^ We can also speculate that, as a result, the average of the four public opinions was not largely different from the participants’ own estimates.

Table S2. Analysis of the initial estimates

Note: The estimates were categorised by size, in comparison to the size of all of the public opinions. We first counted the number of estimates falling into each category for each participant and then totaled the number for all of the participants.

| Category | Frequency in the 20 questions (95% CI) |
| --- | --- |
| Smallest | [2.10, 3.93] |
| Second smallest | [5.68, 7.29] |
| Medium | [5.74, 7.71] |
| Second largest | [2.19, 3.51] |
| Largest | [0.61, 1.35] |

**S6. Additional analysis regarding “When the number of estimates increased”**

As mentioned in the main text, the average of the five estimates was not significantly more accurate than the average of the two estimates. However, the analysis was limited to taking all of the estimates into account. In other words, it is unclear how much the average values become accurate when an individual uses limited estimates (i.e., the number of estimates was 2, 3, and 4).

In order to address this problem, we conducted computer simulations. The procedure was as follows. First, an individual was selected from all of the participants. Second, based on the set of number of estimates, an individual’s estimates were sampled. Note that we assumed that an individual’s own estimate was necessary. In other words, the sampling was conducted among the four simulated estimates. Then, we calculated the mean squared error.

For each value of the number of estimates, we conducted this procedure 10,000 times; Fig. S5 presents the results. As the number of estimates increased, the mean squared error decreased. In other words, as far as this analysis, we could not find a U-shaped relationship between the number of estimates and the mean squared error.


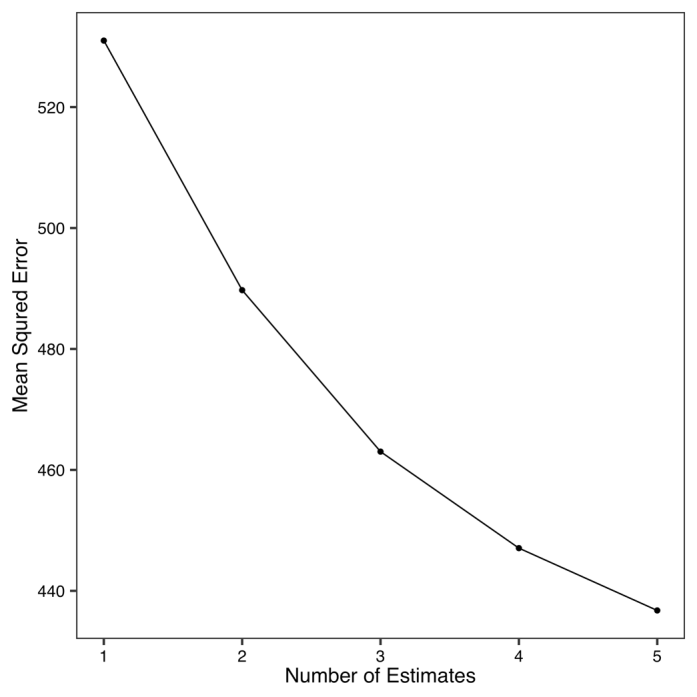


Fig. S5. Results of the additional analysis

Note: When the number of estimates was 2, 3, and 4, we conducted a computer simulation.

**S7. Optimal weighting**

Here, we calculated the optimal weighting of the participants’ own estimate (Estimate 1) and estimate of public opinion (Estimate 2; i.e., the Other’s perspective condition) using the following equation:

(*w* × Estimate 1 + (100–*w*) × Estimate 2)/100

where *w* represents the weighted percentage of a participant’s own estimate. We manipulated *w* from 0 to 100 in steps of 1. Specifically, we set 101 levels (*w* = 0, 1…. .99, and 100) and calculated the MSE for each *w*.

Fig. S7 shows the results indicating the relationship between *w* and the MSE. The red dotted line represents the optimal weighting: In Experiment 1, *w* = 57 and in Experiment 2, *w* = 46. In other words, which estimate should be weighed more depended on the study (Experiment 1: Estimate 1; Experiment 2: Estimate 2).

The figure also shows the dotted gray line for the average estimate (*w* = 50). It can be observed that these MSEs are almost the same to the optimal weighting across the two studies (Experiment 1: red line = 562.44, gray dotted line = 564.02; Experiment 2: red line = 455.00, gray dotted line = 451.54). Subsequently, we can assume that our method (i.e., averaging) is a robust strategy.


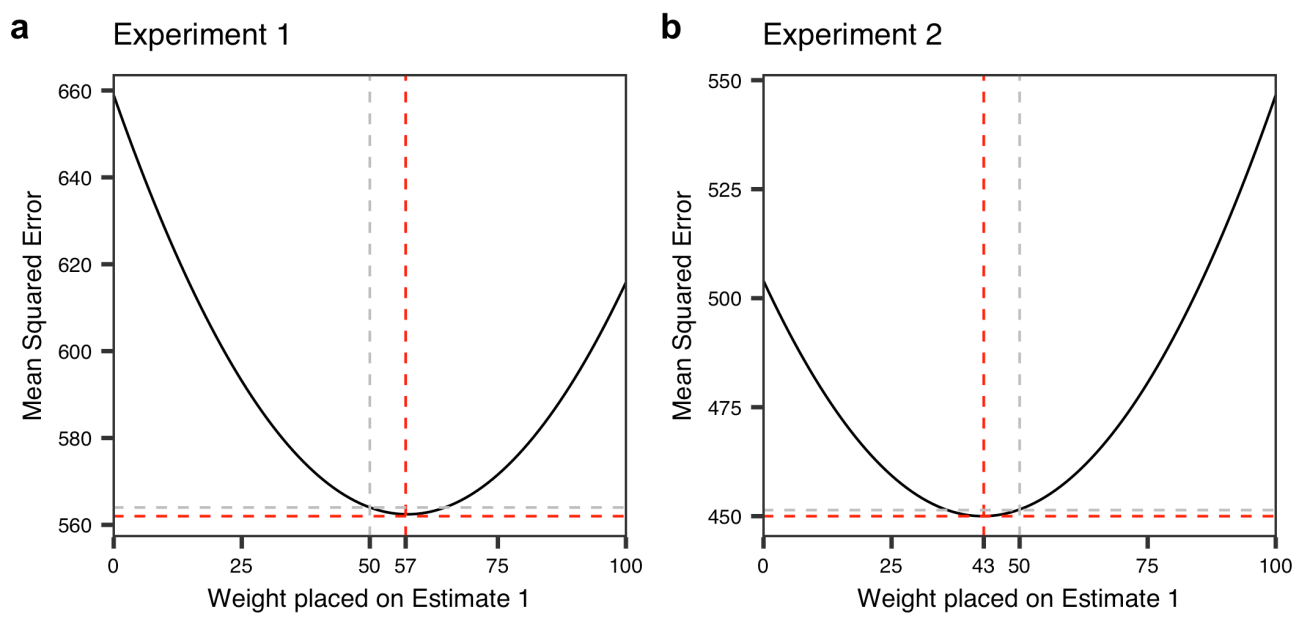


**Fig. S6. Results of optimal weighting in Experiments 1(a) and 2(b).**


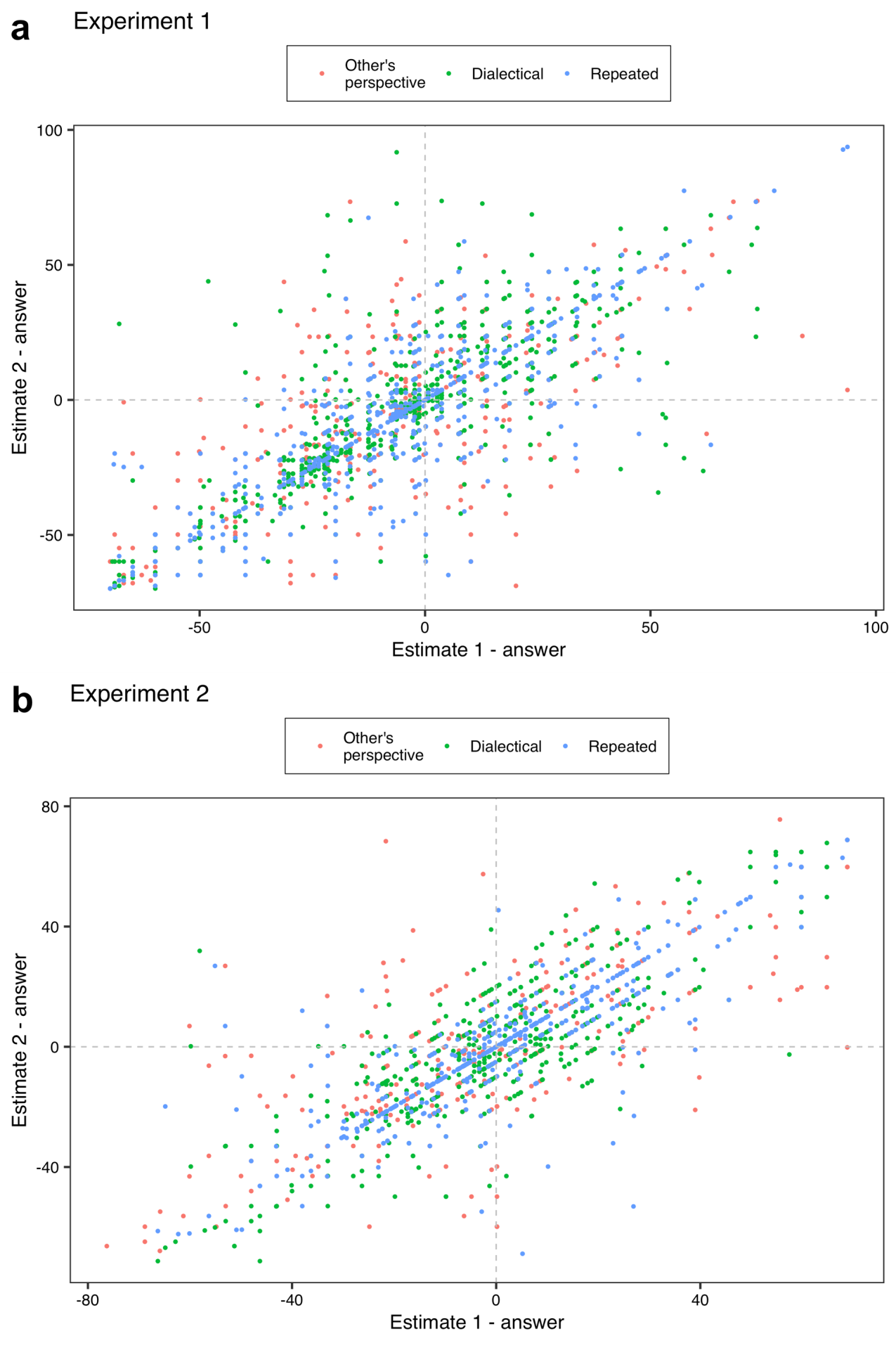
**S8. Results of the estimated value (Analysis 1)**

Fig. S7. Results of the analysis

Note: In this figure, we plotted all of the participants’ estimates.

**Tables S3–S6. Results of the categorisation of the estimates**

**Note: All of the estimates were categorised as overestimate or underestimate, since all of the answers included decimal points. Meanwhile, the estimates were integers. In other words, none of the estimates were the correct answers.**

**Experiment 1**

| **Estimate 1** | Overestimate (%) | Underestimate (%) |
| --- | --- | --- |
| Other’s perspective | 36.42 | 63.58 |
| Dialectical | 35.93 | 64.07 |
| Repeated | 35.76 | 64,24 |

| **Estimate 2** | Overestimate (%) | Underestimate (%) |
| --- | --- | --- |
| Other’s perspective | 37.75 | 62.25 |
| Dialectical | 39.16 | 60.84 |
| Repeated | 35.76 | 64,24 |

**Experiment 2**

| **Estimate 1** | Overestimate (%) | Underestimate (%) |
| --- | --- | --- |
| Other’s perspective | 47.68 | 52.32 |
| Dialectical | 46.30 | 53.70 |
| Repeated | 47.50 | 52.50 |

| **Estimate 2** | Overestimate (%) | Underestimate (%) |
| --- | --- | --- |
| Other’s perspective | 47.32 | 52.68 |
| Dialectical | 47.59 | 52.41 |
| Repeated | 47.50 | 52.50 |

**S8. Results of estimated value (Analysis 2)**


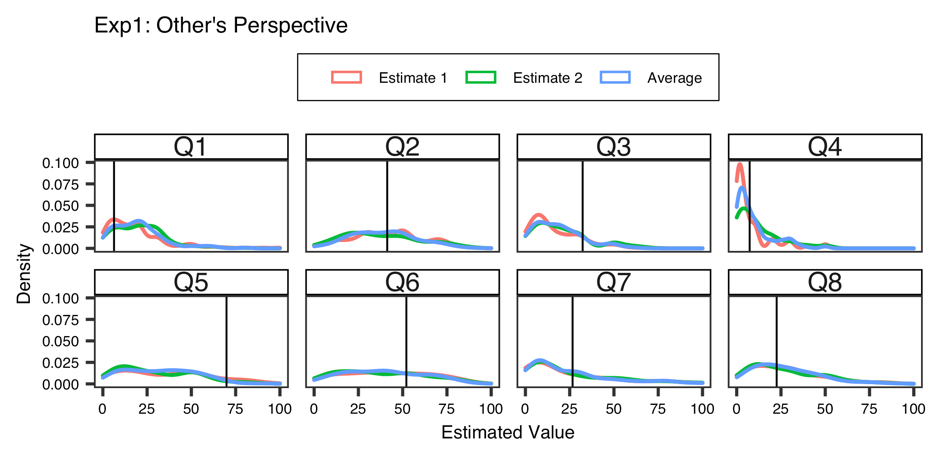


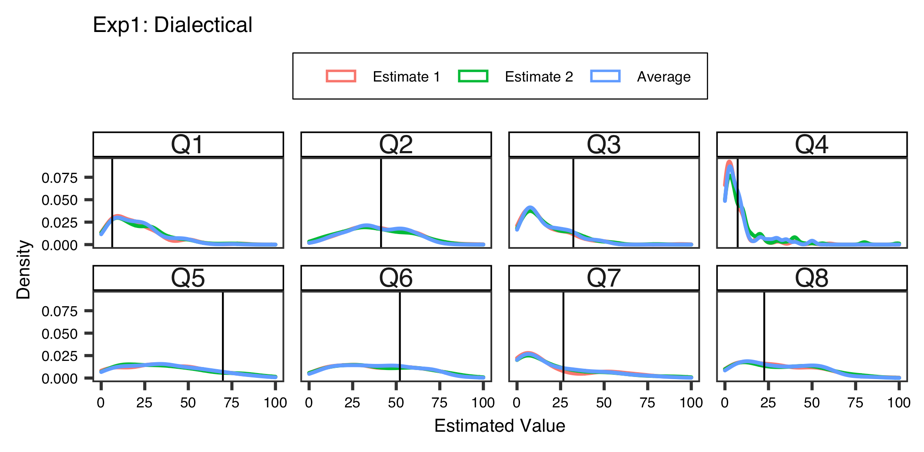
**Fig. S8. Estimated values of the Other’s perspective condition in Experiment 1. Each line represents a probability density function. The black line indicates the correct answer.**


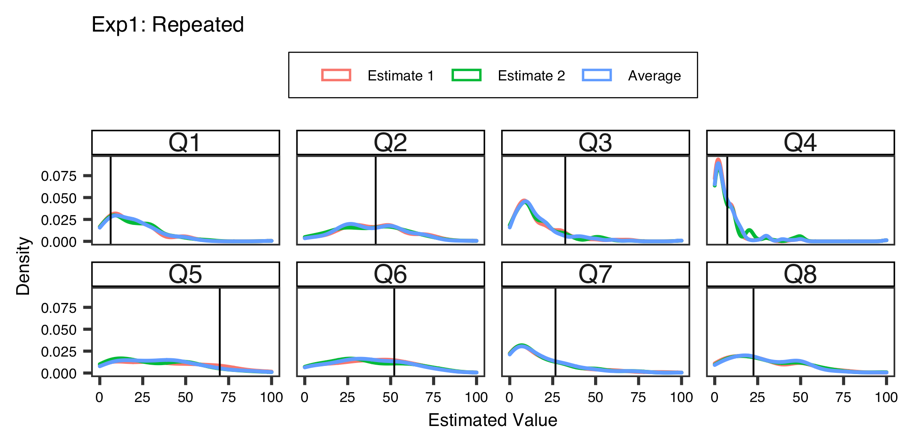
**Fig. S9. Estimated values of the Dialectical condition in Experiment 1.**

**Fig. S10. Estimated values of the Repeated condition in Experiment 1.**


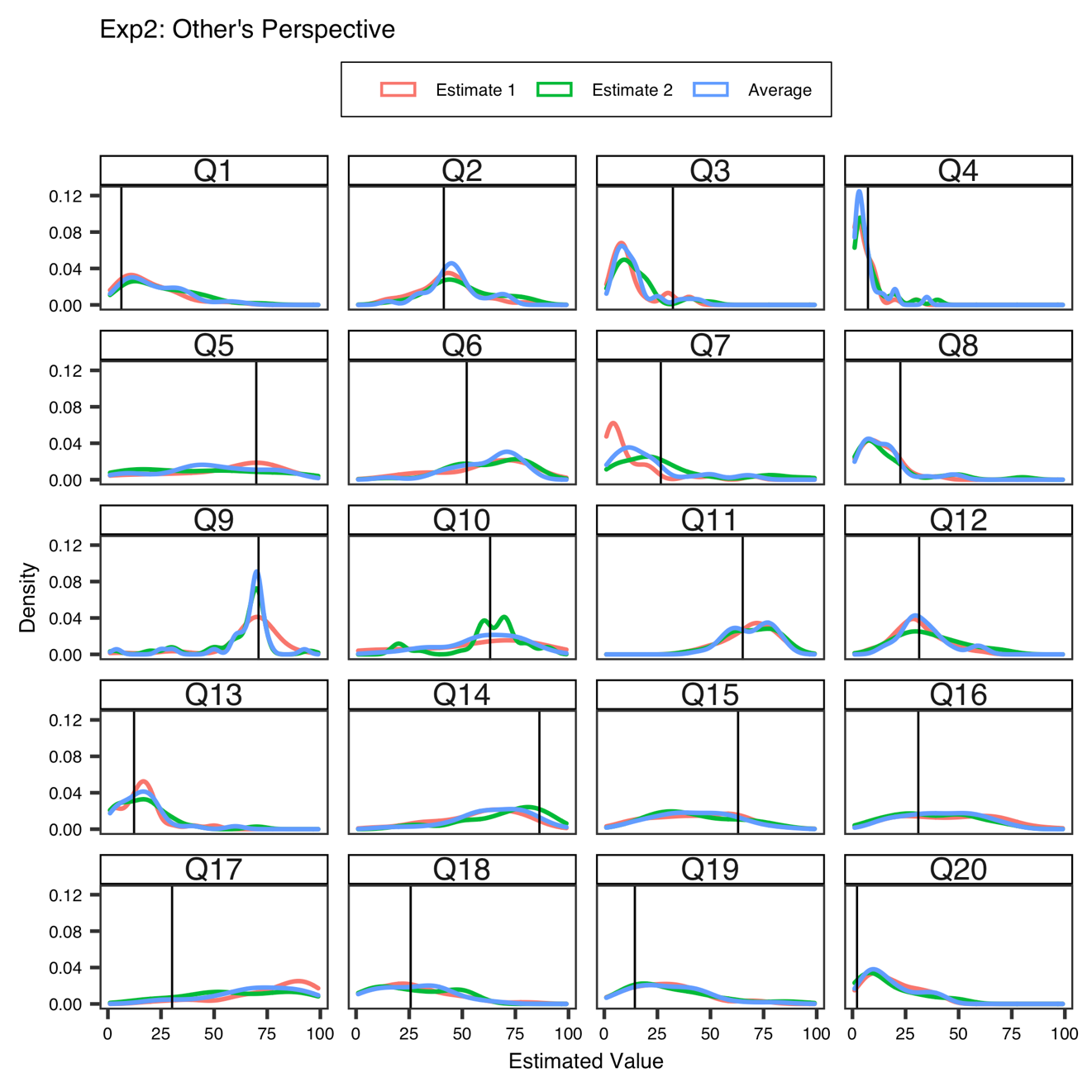


**Fig. S11. Estimated values of the Other’s perspective condition in Experiment 2.**


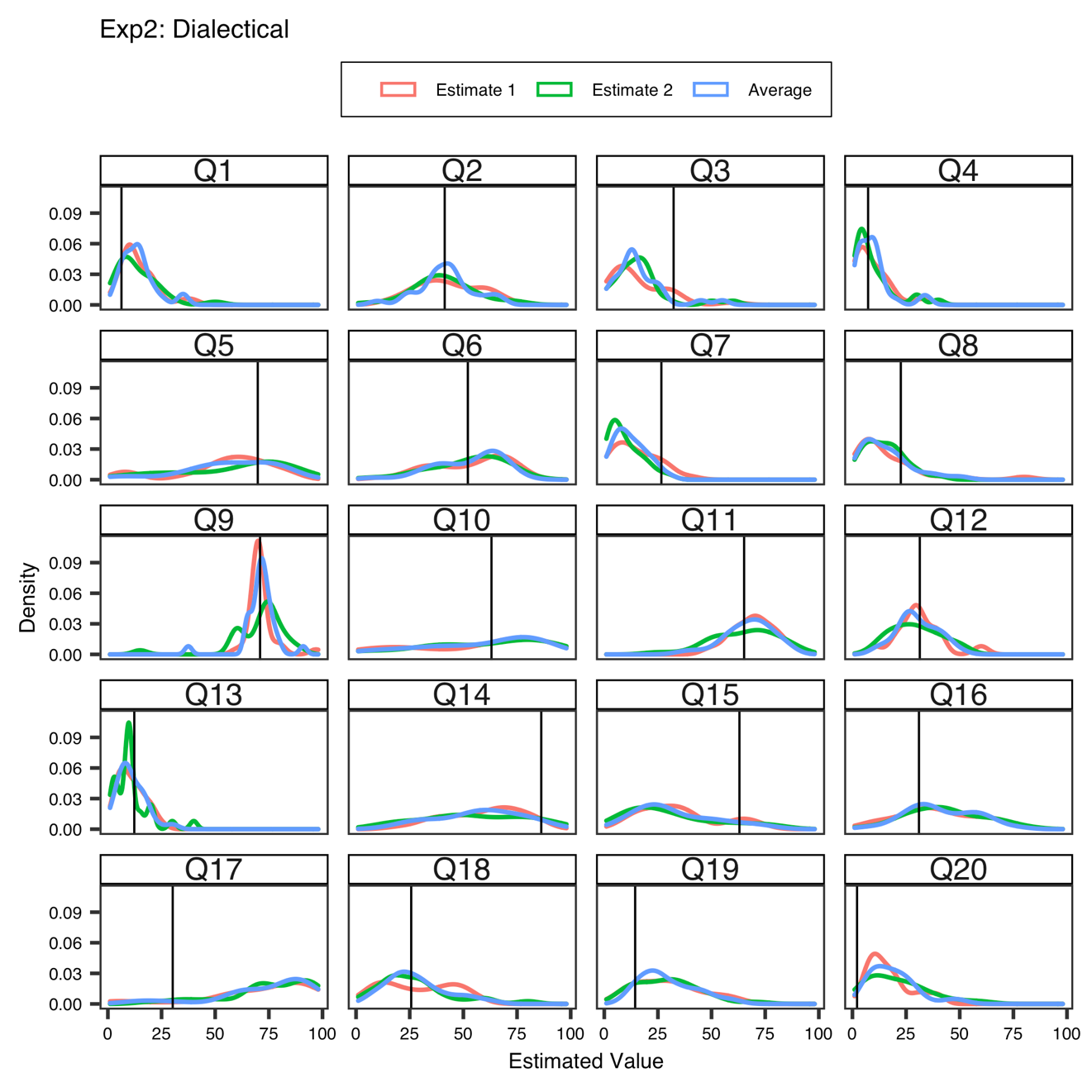


**Fig. S12. Estimated values of the Dialectical condition in Experiment 2.**


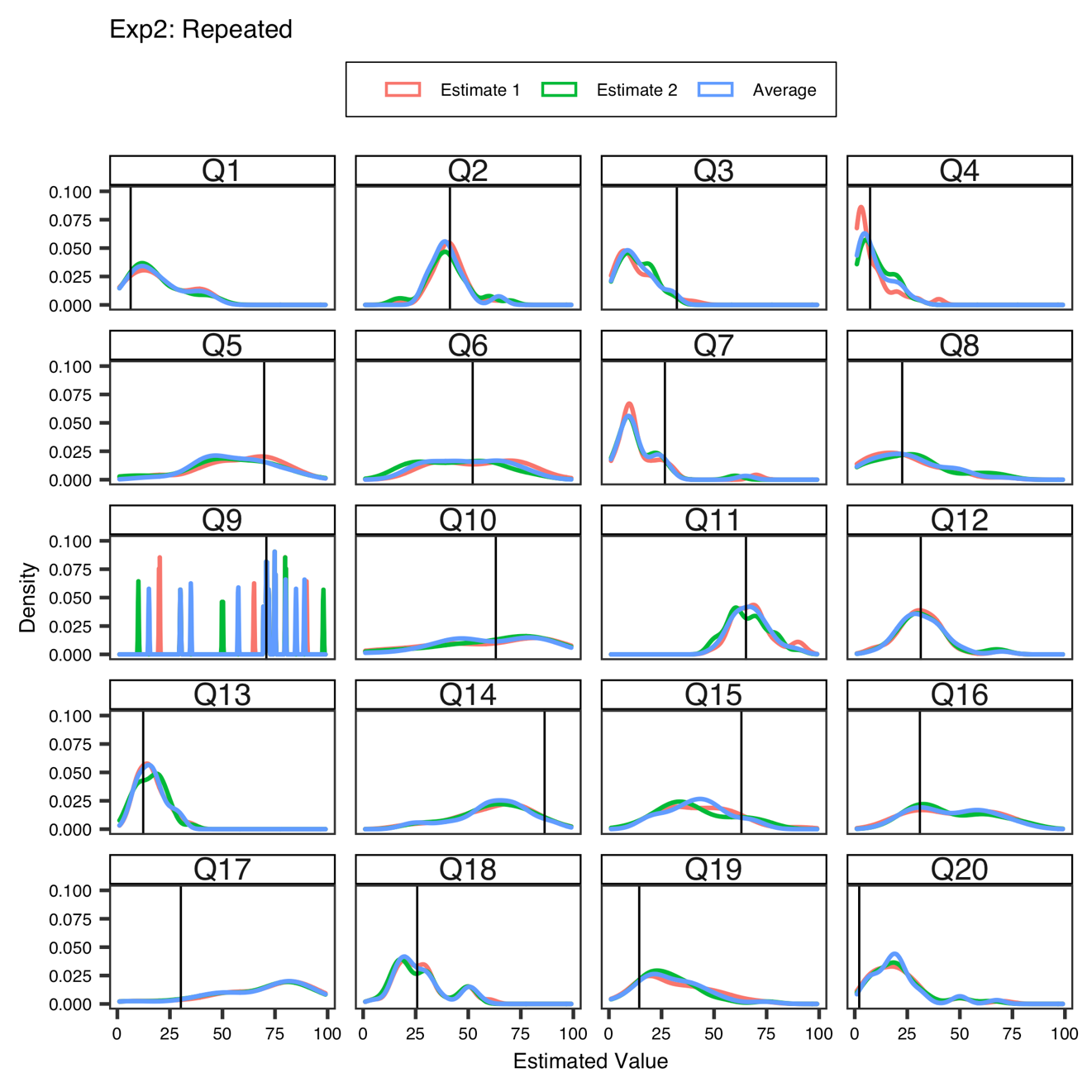


**Fig. S13. Estimated values of the Repeated condition in Experiment 2. In Q9, the estimated values focused on around 75. For this reason, the lines of Estimate 1 and Estimate 2 are hardly visible.**

**References**

1. Strack, F. & Mussweiler, T. Explaining the enigmatic anchoring effect: Mechanisms of selective accessibility. *J. Pers. Soc. Psychol.* **73**, 437–446 (1997).

2. Rader, C. A., Soll, J. B. & Larrick, R. P. Pushing away from representative advice: Advice taking, anchoring, and adjustment. *Organ. Behav. Hum. Decis. Process* **130**, 26–43 (2015). (doi:10.1016/j.obhdp.2015.05.004)

3. Epley, N. & Gilovich, T. The anchoring-and-adjustment heuristic : Why the adjustments are insufficient. *Psychol. Sci.* **17**, 311–318 (2006). (doi:10.1037/0022-3514.82.2.180)
